# Gene-Regulatory Potential of 25-Hydroxyvitamin D_3_ and D_2_

**DOI:** 10.3389/fnut.2022.910601

**Published:** 2022-07-13

**Authors:** Andrea Hanel, Cor Veldhuizen, Carsten Carlberg

**Affiliations:** ^1^Institute of Biomedicine, University of Eastern Finland, Kuopio, Finland; ^2^Carbogen Amcis, B.V., Veenendaal, Netherlands; ^3^Institute of Animal Reproduction and Food Research, Polish Academy of Sciences, Olsztyn, Poland

**Keywords:** vitamin D, transcriptome, PBMCs, target genes, 1α, 25-dihydroxyvitamin D3, 25-hydroxyvitamin D3, 25-hydroxyvitamin D2

## Abstract

Human peripheral blood mononuclear cells (PBMCs) represent a highly responsive primary tissue that is composed of innate and adaptive immune cells. In this study, we compared modulation of the transcriptome of PBMCs by the vitamin D metabolites 25-hydroxyvitamin D_3_ (25(OH)D_3_), 25(OH)D_2_ and 1α,25-dihydroxyvitamin D_3_ (1,25(OH)_2_D_3_). Saturating concentrations of 1,25(OH)_2_D_3_, 25(OH)D_3_ and 25(OH)D_2_ resulted after 24 h stimulation in a comparable number and identity of target genes, but below 250 nM 25(OH)D_3_ and 25(OH)D_2_ were largely insufficient to affect the transcriptome. The average EC_50_ values of 206 common target genes were 322 nM for 25(OH)D_3_ and 295 nM for 25(OH)D_2_ being some 600-fold higher than 0.48 nM for 1,25(OH)_2_D_3_. The type of target gene, such as primary/secondary, direct/indirect or up-/down-regulated, had no significant effect on vitamin D metabolite sensitivity, but individual genes could be classified into high, mid and lower responders. Since the 1α-hydroxylase CYP27B1 is very low expressed in PBMCs and early (4 and 8 h) transcriptome responses to 25(OH)D_3_ and 25(OH)D_2_ were as prominent as to 1,25(OH)_2_D_3_, both vitamin D metabolites may directly control gene expression. In conclusion, at supra-physiological concentrations 25(OH)D_3_ and 25(OH)D_2_ are equally potent in modulating the transcriptome of PBMCs possibly by directly activating the vitamin D receptor.

## Introduction

Although humans can produce vitamin D_3_ endogenously when they expose their skin to UV-B (1), predominant indoor lifestyle as well as insufficient UV indices in Northern latitudes (above 38°) at winter times (2), suggest the supplementation of the vitamin for at least a part of the year (3). Humans and animals use the cholesterol precursor 7-dehydrocholesterol as the substrate for vitamin D_3_ synthesis, while fungi and yeast produce vitamin D_2_ on the basis of the sterol ergosterol (4). Vitamin D_2_ and vitamin D_3_ differ only in the structure of their side chain, but in human intestine the uptake vitamin D_3_ seems to be more effective (5). Nevertheless, both forms of vitamin D are used for food fortification and direct supplementation (6).

Vitamin D_2_ and vitamin D_3_ are metabolized in the liver by the CYP (cytochrome P450) enzymes CYP2R1 and CYP27A1 to 25(OH)D_2_ and 25(OH)D_3_, respectively (7). The sum of the serum concentrations of both metabolites [or sometimes only 25(OH)D_3_] is traditionally used as biomarker for the vitamin D status of an individual (8, 9). A vitamin D status below 50 nM increases the risk for musculoskeletal disorders, such as rickets in children and osteomalacia as well as fractures in adults (10). Moreover, insufficient 25(OH)D serum levels are associated with a number of immune-related diseases, such as rheumatoid arthritis (11), multiple sclerosis (12), type I diabetes (13) and inflammatory bowel disease (14). In addition, vitamin D deficiency raises the risk for severe consequence of microbe infections in tuberculosis, influenza or COVID-19 (coronavirus disease) (15–17). Therefore, one should aim for serum 25(OH)D levels in the range of 75–100 nM, i.e., 30–40 ng/ml (18). In contrast, a vitamin D status of above 250 nM should be avoided, in order to prevent deleterious effects of hypercalcemia (19).

The biologically most active form of vitamin D_3_ and D_2_ are 1,25(OH)_2_D_3_ and 1,25(OH)_2_D_2_, respectively, which function in sub-nanomolar concentrations as nuclear hormones (20). For endocrine purposes 1,25(OH)_2_D is synthesized in the kidneys by the enzyme CYP27B1 using 25(OH)D as a substrate (21), while for paracrine use 1,25(OH)_2_D is produced also in CYP27B1 expressing keratinocytes and immune cells (22). Since 1,25(OH)_2_D is the natural, high affinity (K_D_ = 0.1 nM) ligand of the nuclear receptor VDR (vitamin D receptor) (23, 24), vitamin D affects the activity of hundreds of genes in VDR expressing tissues (25). Thus, the physiological functions of vitamin D are associated with changes of the transcriptome of multiple tissues and cell types by ligand-activated VDR (26). The vitamin D-triggered transcriptome has been studied *in vitro* in a number of cell culture models, such as THP-1 monocytic leukemia cells (27–29), as well as in PBMCs (30, 31). Primary cells like PBMCs are a natural mixture of innate and adaptive immune cells like monocytes, natural killer cells, T and B cells. They are far closer to the human *in vivo* situation than cell lines and can be obtained with minimal harm to the donor (32).

The affinity of VDR for 25(OH)D_3_ is 100- to 1,000-fold lower than for 1,25(OH)_2_D_3_ (33, 34), which parallels with the fact that the serum concentration of 25(OH)D_3_ is some 1,000-fold higher than that of 1,25(OH)_2_D_3_ (0.05–0.15 nM) (35). This relation raised already earlier the question, whether 25(OH)D_3_ has the potential to act as a nuclear hormone that directly activates the VDR (36). The molecule 1,25(OH)_2_D_3_ has three hydroxyl groups, each of which is specifically contacted by a pair of polar amino acids within VDR's ligand-binding pocket (37, 38). In contrast, 25(OH)D lacks the hydroxyl group at carbon 1 and therefore binds with lower affinity to the receptor, but takes the same agonistic conformation within the ligand-binding pocket (39).

In this study, we analyzed the transcriptome-wide effects of 25(OH)D_3_ and 25(OH)D_2_ in comparison to that of 1,25(OH)_2_D_3_ in freshly isolated human PBMCs. We will demonstrate that 25(OH)D_3_ and 25(OH)D_2_ are equally potent in modulating the transcriptome of PBMCs and cannot exclude the possibility that both vitamin D metabolites directly activate the VDR.

## Materials and Methods

### Sample Collection

Peripheral blood was collected from a single healthy individual (male, age 57 years) the vitamin D responsiveness of whom had already been characterized in the VitDbol trial (NCT02063334) (32, 40). The ethics committee of the Northern Savo Hospital District had approved the study protocol (#9/2014). The individual gave written informed consent to participate in the study and the experiments were performed in accordance with relevant guidelines and regulations.

### PBMC Isolation and Stimulation

PBMCs were isolated immediately after collecting 100 ml peripheral blood using Vacutainer CPT Cell Preparation Tubes with sodium citrate (Becton Dickinson) according to manufacturer's instructions. After washing with phosphate-buffered saline, the cells were grown at a density of 0.5 million/ml in 5 ml RPMI1640 medium supplemented with 10% charcoal-depleted fetal calf serum, 2 mM L-glutamine, 0.1 mg/ml streptomycin and 100 U/ml penicillin at 37 °C in a humidified 95% air/5% CO_2_ incubator and treated for 4, 8 or 24 h either with 1,25(OH)_2_D_3_ (0.1, 1 and 10 nM) 25(OH)D_3_ (100, 250, 500, 750, and 1,000 nM), 25(OH)D_2_ (100, 250, 500, 750, and 1,000 nM) or solvent (0.1% EtOH). All experiments were performed in three repeats. Deconvolution of RNA-seq data using the algorithm CIBERSORTx (41) and its LM6 signature matrix estimated the relative amounts of B cells (7%), CD8^+^ T cells (32%), CD4^+^ T cells (20%), natural killer cells (21%) and monocytes/macrophages (20%) within the PBMC pool.

### RNA-Seq Data Generation and Processing

Total RNA was isolated using the High Pure RNA Isolation Kit (Roche) according to manufacturer's instructions. RNA quality was assessed on an Agilent 2100 Bioanalyzer system (RNA integrity number ≥ 8). rRNA depletion and cDNA library preparation were performed using the New England Biolabs kits NEBNext rRNA Depletion, NEBNext Ultra II Directional RNA Library Prep for Illumina and NEBNext Multiplex Oligos for Illumina (Index Primers Sets 1 and 2) according to manufacturer's protocols. RNA-seq libraries went through quality control on an Agilent 2100 Bioanalyzer and were sequenced on a NextSeq 500 system (Illumina) at 75 bp read length using standard protocols at the Gene Core facility of the EMBL (Heidelberg, Germany). The libraries were sequenced as four batches. Fastq files of the 66 libraries can be found at Gene Expression Omnibus (GEO, www.ncbi.nlm.nih.gov/geo) with accession number GSE199273.

Single-end, reverse-stranded cDNA sequence reads were aligned to the reference genome (version GRCh38) with Ensembl annotation (version 103) by using default settings of the nf-core/rnaseq STAR-Salmon pipeline (version 3.0) (http://doi.org/10.5281/zenodo.4323183) (42). The number of nucleotide sequence reads are shown in Supplementary Table 1. Ensembl gene identifiers were annotated with gene symbol, description, genomic location and biotype by accessing the Ensembl database (version 104) *via* the R package *BiomaRt* (version 2.46.0) (43). Ensembl gene identifiers missing HGNC gene symbol annotation, Entrez ID, genomic location information or being mitochondrially encoded were removed from the datasets. When a gene name appeared more than once, the entry with the highest average gene counts was kept.

### Transcriptome Data Analysis

**Figure 1:**
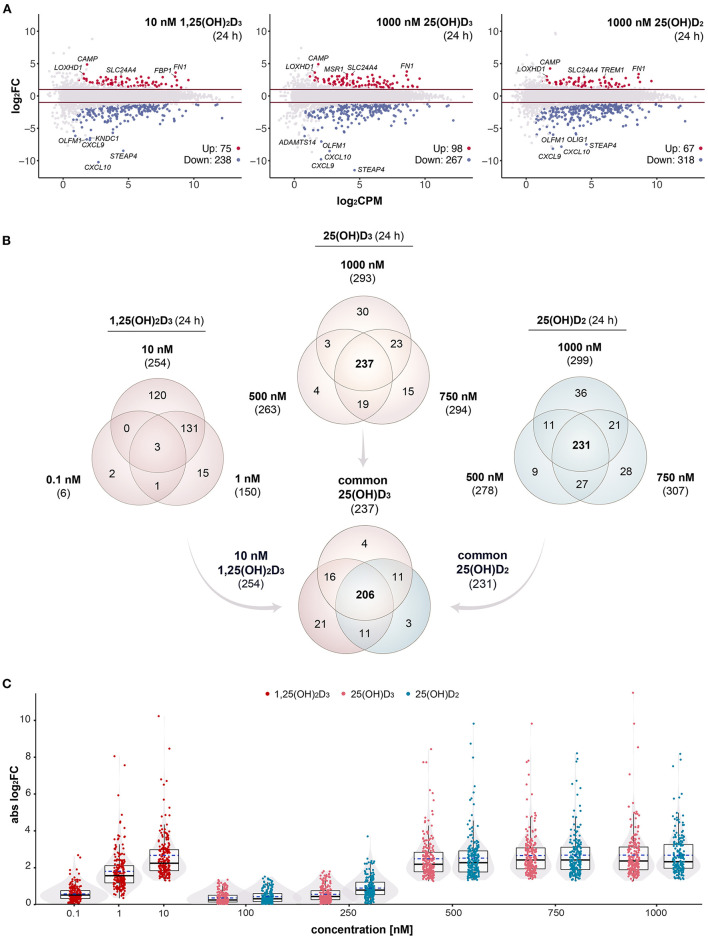
Gene-regulatory potential of vitamin D metabolites. MA plots display the genome-wide transcriptional response of PBMCs treated for 24 h with 10 nM 1,25(OH)_2_D_3_, 1000 nM 25(OH)D_3_ or 1000 nM 25(OH)D_2_ in comparison to solvent (0.1% EtOH) **(A)**. For each gene, the change of expression (log_2_FC) between treated and control samples is shown in relation to its mean expression level (log_2_CPM). Differential expression analysis was performed as a pairwise comparison per each concentration by using *glmTreat* test. Significantly (FDR < 0.05) up- and down-regulated genes are highlighted in red and blue, respectively. Horizonal lines (red) indicate the applied statistical testing threshold (absolute FC > 2). The top 5 responsive genes (up- and down-regulated) are labeled. Venn diagrams show the overlap of vitamin D target genes per metabolite **(B)**. The relations between all treatments and concentrations (at 24 h) are provided in Supplementary Figure 3. Box and violin plots summarize the distribution of the magnitude of expression change (absolute log_2_FC) of the 206 common genes for each vitamin D metabolite and concentration **(C)**. Solid lines within the boxes indicate medians, while dashed lines mark the mean.

Differential gene expression analysis was computed in R (version 4.1.2) in the Rocky Linux 8.5 operating system using the tool *EdgeR* (version 3.36.0) (44). The analysis focused on the 19,142 protein-coding genes, in order to reduce transcriptional noise expected by non-coding genes. Read counts were normalized for differences in library size to counts per million (CPM). Genes with very low expression were filtered out by applying the function *FilterByExpr()*, in order to mitigate the multiple testing problem and to not interfere with the statistical approximations of the *EdgeR* pipeline. This requirement was fulfilled by 12,939 genes. After filtering, library sizes were recomputed and trimmed mean of M-value normalization was applied. The transcriptome data structure was explored *via* the dimensionality reduction method multidimensional scaling (MDS) (Supplementary Figure 1). MDS was computed *via EdgeR*'s function *plotMDS()*, in which distances approximate the typical log_2_ fold change (FC) between the samples. This distance was calculated as the root mean square deviation (Euclidean distance) of the largest 500 log_2_FCs between a given pair of samples, i.e., for each pair a different set of top genes was selected. The inspection of the MDS plot showed that (i) the time-dependent divergence from the native transcriptome state and (ii) the concentration-dependent modulation by treatment with 1,25(OH)_2_D_3_, 25(OH)D_3_ or 25(OH)D_2_ are the two principal factors driving differences in the gene expression profiles of PBMCs. The gene-wise statistical test for differential expression was computed using the generalized linear model quasi-likelihood pipeline (45). The empirical Bayes shrinkage was robustified against outlier dispersions as recommended (45). The *glmTreat* approach was used to test for differential expression relative to FC > 1.5 at the early time points (4 and 8 h) and FC > 2 at 24 h. Taking all treatments together, 553 genes with a Benjamini-Hochberg corrected *p*-value [i.e., false discovery rate (FDR)] < 0.05 and a total trimmed mean of M-value normalized CPM count > 10 (i.e., the sum of the average gene expression level of the reference 10 nM 1,25(OH)_2_D_3_ and EtOH-treated samples at each time point) were considered as vitamin D targets (Supplementary Table 2). Mean-Difference (MA) plots (Figures 1A, **3A**; Supplementary Figure 2) for the 19 different treatment conditions were generated with *vizzy* (version 1.0.0, https://github.com/ATpoint/vizzy).

### Dose Response Analysis

The effect of vitamin D metabolites on the change in mRNA levels (absolute log_2_FC) was modeled with the three-parameter log-logistic function LL.3 from the R package *drc* (46) having lower limit fixed at 0. The estimated relative EC_50_ values and their standard errors were retrieved from the curve fits *via* the *summary()* function. The quality of fitting was checked by manual inspection. EC_50_ values were reported only for those 206 genes, for which the value could be estimated for all three vitamin D metabolites. Pairwise comparisons of EC_50_ values between different metabolites as well as groups of target genes were performed using Tukey's test implemented in the R package *multcomp* (version 1.4.18) and family-wise error rate (FWER)-adjusted *p*-values retrieved. Comparisons with a FWER < 0.05 were considered as significant. The code of the analysis can be found at https://github.com/andreahanel/2022_Doseresponse.

## Results

### Transcriptome-Wide Responses to 1,25(OH)_2_D_3_, 25(OH)D_3_, and 25(OH)D_2_

In order to obtain maximal responses of the transcriptome, PBMCs from an healthy individual were treated immediately after isolation for 24 h with either 10 nM 1,25(OH)_2_D_3_, 1,000 nM 25(OH)D_3_, 1,000 nM 25(OH)D_2_ or solvent (0.1% EtOH). In three repeats RNA-seq was performed on the basis of total RNA. As in comparable studies (31, 47, 48), strict statistical thresholds of FDR < 0.05 and FC > 2 were applied. This resulted in 313 genes (75 up-regulated, 238 down-regulated) responding to 1,25(OH)_2_D_3_, 365 target genes of 25(OH)D_3_ (98 up-regulated, 267 down-regulated) and 385 genes modulated by 25(OH)D_2_ (67 up-regulated, 318 down-regulated) (Figure 1A). For all three vitamin D metabolites the genes *CAMP* (cathelicidin antimicrobial peptide), *FN1* (fibronectin 1), *LOXHD1* (lipoxygenase homology PLAT domains 1) and *SLC24A4* (solute carrier family 24 member 4) were the top up-regulated and the genes *CXCL10* (C-X-C motif chemokine ligand 10), *STEAP4* (STEAP4 metalloreductase), *CXCL9* and *OLFM1* (olfactomedin 1) the most down-regulated.

In addition to saturating ligand concentrations, PBMCs were treated for 24 h with 0.1 and 1 nM 1,25(OH)_2_D_3_, with 100, 250, 500, and 750 nM 25(OH)D_3_ as well as with 100, 250, 500, and 750 nM 25(OH)D_2_. This resulted in 7 and 179 target genes for 0.1 and 1 nM 1,25(OH)_2_D_3_, no targets for 100 and 250 nM 25(OH)D_3_, 322 and 392 responding genes for 500 and 750 nM 25(OH)D_3_, no targets for 100 nM 25(OH)D_2_ as well as 30, 342, and 397 genes that reacted on a stimulation with 250, 500, and 750 nM 25(OH)D_2_ (Supplementary Figure 3A). These in total 526 different genes were filtered with a list of 662 genes, which were detected by time course analysis of treatment of PBMCs with 1,25(OH)_2_D_3_ (31). This led to 389 confirmed vitamin D target genes, of which 6, 150, and 254 responded to 0.1, 1 and 10 nM 1,25(OH)_2_D_3_, 263, 294, and 293 to 500, 750, and 1,000 nM 25(OH)D_3_ as well as 25, 278, 307, and 299 to 250, 500, 750, and 1,000 nM 25(OH)D_2_ (Supplementary Figure 3B). Venn diagrams indicated that there are 237 common targets of 500, 750, and 1,000 nM 25(OH)D_3_ as well as 231 common targets of 500, 750, and 1,000 nM 25(OH)D_2_ (Figure 1B). From both lists 206 genes are identical with the 254 targets responding to 10 nM 1,25(OH)_2_D_3_.

The 206 common targets represent a stable set of genes responding to variant concentrations of all three tested vitamin D metabolites (Figure 1C). Combined box plots and violin plots monitored the overall change in the expression of these 206 genes with raising metabolite concentrations. Interestingly, 250 nM of both 25(OH)D_3_ and 25(OH)D_2_ were insufficient for general gene regulation, while 500 nM of both vitamin D metabolites was clearly above this threshold.

In summary, both by number as well as by most responsive target genes the transcriptome-wide responses to saturating concentrations of 1,25(OH)_2_D_3_, 25(OH)D_3_ and 25(OH)D_2_ are very comparable. At concentrations of 250 nM and below, 25(OH)D_3_ and 25(OH)D_2_ are largely insufficient to significantly modulate the expression of vitamin D target genes.

### Gene-Specific Sensitivity to Vitamin D Metabolites

**Figure 2:**
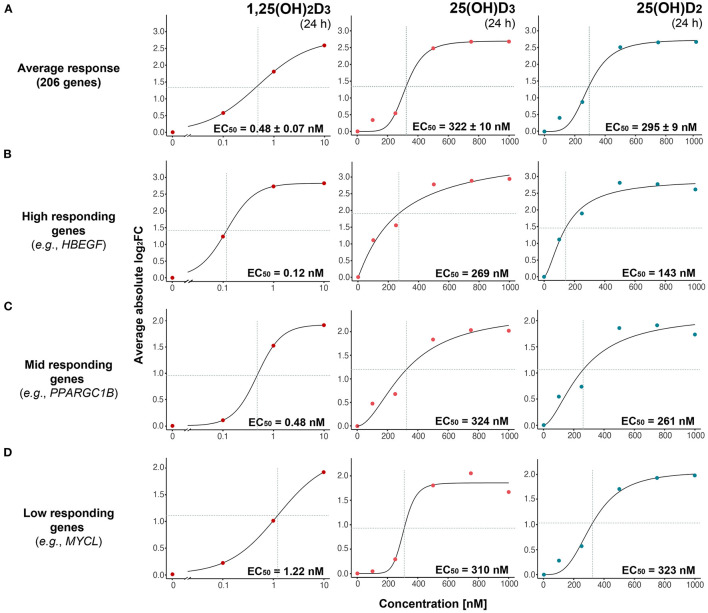
Gene-specific response to vitamin D metabolites. The magnitude of expression change (absolute log_2_FC) as a function of vitamin D metabolite concentration is modeled with a three-parameter log-logistic model based on the average of 206 common targets **(A)** or indicated individual genes **(B–D)**. Genes are segregated into high-, mid- and low-responding based on their estimated EC_50_ values in response to 1,25(OH)_2_D_3_. Standard errors of the EC_50_ estimates are indicated.

Plotting the FC of the 206 common vitamin D target genes over vitamin D metabolite concentration and using the three-parameter log-logistic model, determined the EC_50_ values 0.48 nM for 1,25(OH)_2_D_3_, 322 nM for 25(OH)D_3_ and 295 nM for 25(OH)D_2_ (Figure 2A). There is statistically no significant difference (*FWER* > 0.05, Tukey's test) between the potencies of 25(OH)D_3_ and 25(OH)D_2_, but their average EC_50_ is some 640-fold higher than that of 1,25(OH)_2_D_3_.

Based on the reference dataset (31), the 206 common targets were subdivided either into 85 primary targets (25 up- and 60 down-regulated) and 121 secondary targets (17 up- and 104 down-regulated) or into 94 direct targets (31 up- and 63 down-regulated) and 112 indirect targets (11 up- and 101 down-regulated). For 1,25(OH)_2_D_3_ the EC_50_-values of the eight different categories varied from 0.29 to 0.73 nM but did not differ significantly (*FWER* > 0.05, Tukey's test) between each other (Supplementary Figure 4). Similarly, for 25(OH)D_3_ the range was 318 to 389 nM and for 25(OH)D_2_ 192 to 313 nM, but the difference was not statistically significant (*FWER* > 0.05, Tukey's test).

Since neither gene categories nor up- or down-regulation allowed a distinction of the sensitivity of vitamin D target genes to vitamin D metabolites, the dose responses of the 206 genes were investigated on an individual gene level. Manual inspection of the dose response curves provided for 130 genes convincing fits for all three vitamin D metabolites. Interestingly, for 1,25(OH)_2_D_3_ the EC_50_-values ranged from 0.10 nM [*ENPP2* (ectonucleotide pyrophosphatase/phosphodiesterase 2)] to 2.39 nM [*LMNA* (lamin A/C)], for 25(OH)D_3_ from 121 nM [*NXPH4* (neurexophilin 4)] to 461 nM [*ENTPD7* (ectonucleoside triphosphate diphosphohydrolase 7)] and for 25(OH)D_2_ from 132 nM (*SLC11A1*) to 421 nM [*STAB1* (stabilin 1)] (Supplementary Table 2). The wide range of gene-specific sensitivity to 1,25(OH)_2_D_3_ allowed the categorization of the representative 130 vitamin target genes into 59 high responders (EC_50_ value range from 0.10 to 0.39 nM), 59 mid responders (0.41 to 1.06 nM) and 12 low responders (1.20 to 2.39 nM). Representative genes for the three categories are *HBEGF* (heparin binding EGF like growth factor) as high responding gene (Figure 2B), *PPARGC1B* (PPARG coactivator 1 beta) as mid responding gene (Figure 2C) and *MYCL* (MYCL proto-oncogene, BHLH transcription factor) as low responding gene (Figure 2D). Importantly, the far smaller range of the gene-specific EC_50_ values for 25(OH)D_3_ and 25(OH)D_2_ did not allow a categorization of the vitamin D target genes by their response to these metabolites.

Taken together, the average EC_50_ values of the response of vitamin D target genes to 25(OH)D_3_ and 25(OH)D_2_ are not significantly different and are in the order of 300 nM, i.e., some 600-fold higher as those for 1,25(OH)_2_D_3_. Categorization of the target genes into primary/secondary, direct/indirect or up-regulated/down-regulated does not allow any significant distinction in their response to the three vitamin D metabolites. However, individual target genes can be classified by their response to 1,25(OH)_2_D_3_ (but not to 25(OH)D_3_ and 25(OH)D_2_) as high, mid and low responding genes.

### Time Course Response of Vitamin D Target Genes

**Figure 3:**
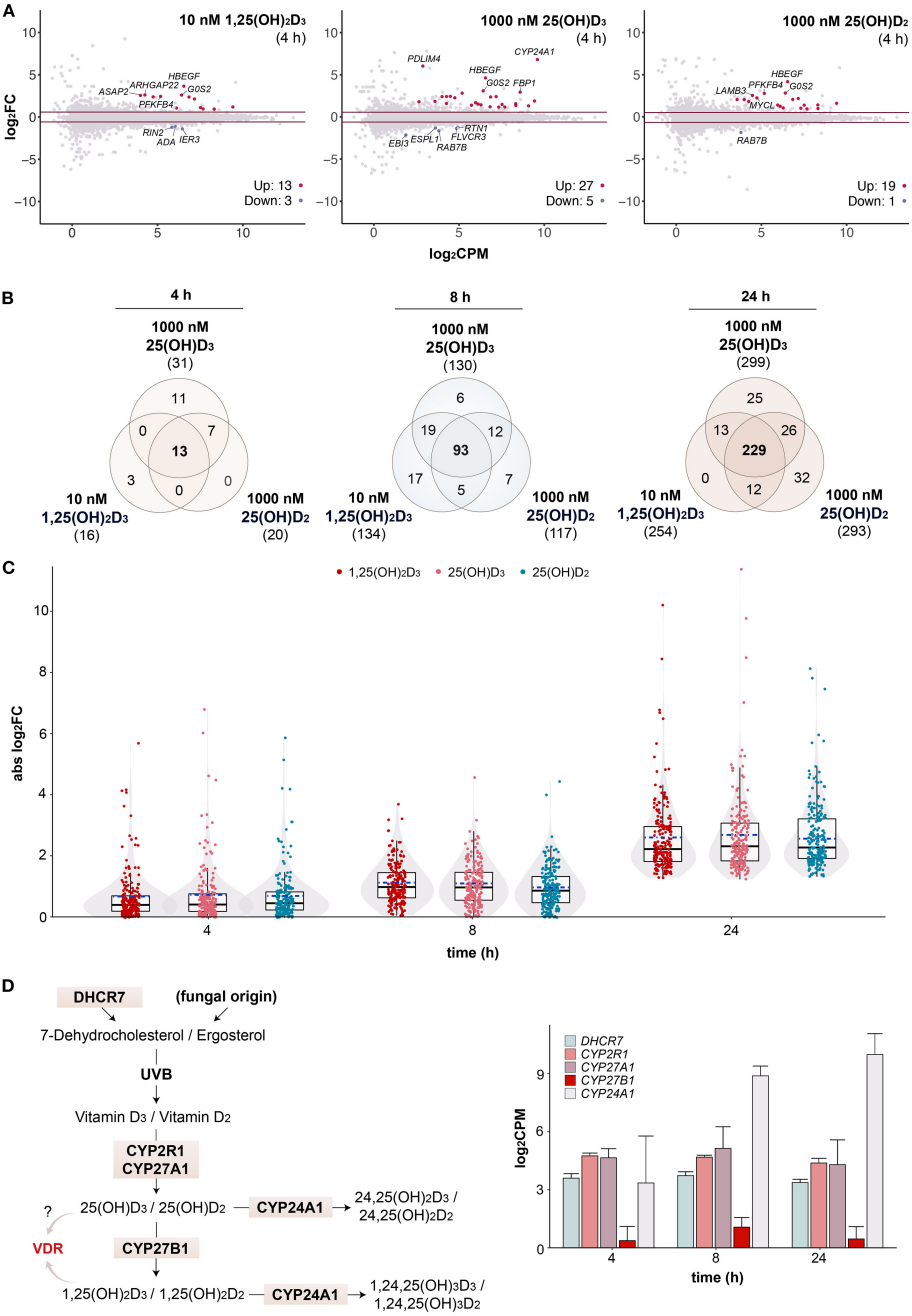
Changes in transcriptional profiles over time. MA plots show early gene expression changes in PBMCs treated for 4 h with 10 nM 1,25(OH)_2_D_3_, 1000 nM 25(OH)D_3_ or 1000 nM 25(OH)D_2_ in comparison to solvent (0.1% EtOH, i.e., time-matched control) **(A)**. For each gene, difference in expression (log_2_FC) between treated and control samples is shown in relation to its mean expression level (log_2_CPM). Genes were tested for differential expression relative to an absolute FC > 1.5 using *glmTreat* method. Horizonal lines (red) indicate the applied statistical testing threshold. The top 5 most responsive up- and down regulated genes (if any; FDR < 0.05) are highlighted. Venn diagrams show the overlap of vitamin D target genes per time point **(B)**. The relations between all treatments are provided in Supplementary Figure 5. Box and violin plots summarize the distribution of the magnitude of expression change (absolute log_2_FC) of the 206 common genes for each vitamin D metabolite and time point **(C)**. Solid lines within the boxes indicate medians, while dashed lines mark the mean. Please note that the data of the 24 h time point serve as a reference and are identical to those presented in Figure 1C. A map of the human vitamin D metabolism pathway marks key enzymes [**(D), left**]. The mean of 1,25(OH)_2_D_3_-treated and untreated mRNA expression of the five indicated genes is displayed in log_2_-scale as columns for all three time points [**(D), right**]. Error bars indicate standard deviation.

In order to investigate whether 25(OH)D_3_ and 25(OH)D_2_ may activate vitamin D signaling without enzymatic conversion by CYP27B1 to 1,25(OH)_2_D_3_ and 1,25(OH)_2_D_2_, respectively, the transcriptome-wide response to saturating concentrations of all three vitamin D metabolites was assessed by RNA-seq at earlier time points. After 4 h stimulation with 10 nM 1,25(OH)_2_D_3_, 16 genes (13 up- and 3 down-regulated) passed the statistical threshold (FDR < 0.05, FC > 1.5), with 1,000 nM 25(OH)D_3_ 32 genes (27 up- and 5 down-regulated) and with 1,000 nM 25(OH)D_2_ 20 genes (19 up- and 1 down-regulated) (Figure 3A). Common top up-regulated genes were *HBEGF* and *G0S2* (G0/G1 switch 2). After filtering with the reference dataset of vitamin D target genes in PBMCs (Supplementary Figure 5) (31), Venn diagrams indicated that there are 13 common genes (out of 31 in total) of the three vitamin D metabolites responding already after 4 h, 93 (out of 159 in total) after 8 h and 229 (out of 337 in total) after 24 h (Figure 3B). As observed in previous time course studies (29, 31), at earlier time points there are more up- than down-regulated genes, since the process of up-regulation is more straightforward and quicker than that of down-regulation. When comparing the response of the 206 common target genes to the three vitamin D metabolites, there was no significant difference in the response to 10 nM 1,25(OH)_2_D_3_, 1,000 nM 25(OH)D_3_ or 1,000 nM 25(OH)D_2_ at 4, 8 and 24 h (Figure 3C).

The enzymes DHCR7 (7-dehydrocholesterol reductase), CYP2R1, CYP27A1, CYP27B1 and CYP24A1 are critical nodes in the vitamin D metabolism pathway (Figure 3D, left). Therefore, the relative mRNA expression of the genes encoding for these enzymes were extracted from the transcriptome datasets and compared at the time points 4, 8 and 24 h (Figure 3D, right). *DHCR7, CYP2R1* and *CYP27A1* show comparable mid-range expression, while the average expression of *CYP27B1* is 10- to 32-fold lower. In this way, *CYP27B1* belongs to the 5% lowest expressed genes in PBMCs. Since the *CYP24A1* gene is a well-known vitamin D target gene (49), its expression is even 671-fold higher than that of *CYP27B1*. For comparison, the relative expression values of all five genes in PBMCs of 12 participants of our VitDHiD study (50) are displayed (Supplementary Figure 6). The individuals were ranked by increasing *CYP27B1* expression, which varied by a factor of 12.5, but being in average some 200-fold lower as the *CYP24A1* expression. Thus, in vitamin D-triggered PBMCs the synthesis of 1,25(OH)_2_D on the basis of 25(OH)D is far less prominently covered by enzyme expression as the degradation of 25(OH)D and 1,25(OH)_2_D to 24,25(OH)D and 1,24,25(OH)_3_D, respectively.

In summary, already at early time points (4 and 8 h) the PBMC transcriptome responds to a stimulation with saturating concentrations of 25(OH)D_3_ and 25(OH)D_2_ as prominently as to 1,25(OH)_2_D_3_. The expression of CYP27B1 protein in PBMCs is very likely too low for an efficient conversion of 25(OH)D_3_ and 25(OH)D_2_ into 1,25(OH)_2_D_3_ and 1,25(OH)_2_D_2_, respectively, in particular in the presence of highly expressed CYP24A1 protein. However, it cannot be excluded that metabolic formation of 1,25(OH)_2_D is partly contributing to the results obtained.

## Discussion

The focus of this study was to compare the gene regulatory potential of the vitamin D metabolites 25(OH)D_3_ and 25(OH)D_2_ to each other and in reference to 1,25(OH)_2_D_3_. At supra-physiological concentrations of 10 nM 1,25(OH)_2_D_3_ (100-times the natural serum concentration) and 1,000 nM 25(OH)D (10-times the recommended serum level) the response of the PBMC transcriptome is very comparable, i.e., the expression of some 300 genes is significantly modulated showing after 24 h stimulation some 3-times more down-regulation than up-regulation. Moreover, the target gene lists of 25(OH)D_3_, 25(OH)D_2_ and 1,25(OH)_2_D_3_ are to 85% identical, i.e., our experimental series had relatively low transcriptional noise. These observations suggest that all three vitamin D metabolites use identical mechanisms in the modulation of vitamin D target gene expression. Form a structural point of view this is obvious, since 25(OH)D will bind in the same agonistic VDR conformation as 1,25(OH)_2_D (51).

The 206 common vitamin D target genes, on which we focus in this study, represent the majority of the vitamin D sensitive part of the PBMC transcriptome, although (in order to further reduce transcriptional noise) they had been filtered by a reference dataset from a recent 1,25(OH)_2_D_3_ time course study in PBMCs (31). The estimation of average EC_50_ values of 322 nM for 25(OH)D_3_ and 295 nM for 25(OH)D_2_ compared to 0.48 nM for 1,25(OH)_2_D_3_ is the first report on the sensitivity of the transcriptome to vitamin D metabolites. These results provide an additional argument that there is no difference in the response of the transcriptome to 25(OH)D_3_ and 25(OH)D_2_. Moreover, our findings indicate with the factor of ~600 a good estimation of the relative gene regulatory potential of 25(OH)D compared to 1,25(OH)_2_D. Since serum levels of 25(OH)D are even 1,000-times higher than that of 1,25(OH)_2_D, this supports the option that 25(OH)D can directly modulate the expression of vitamin D target genes. However, 25(OH)D concentrations in the order of 300 nM are far higher than the recommended 100 nM. Therefore, irrespective of the mechanism of action, for persons with a normal vitamin D status the transcriptome as a whole may not be regulated by 25(OH)D. However, there are a few very sensitive genes, such as *NXPH4, SLC11A1, ADGR3* (adhesion G protein-coupled receptor G3), *G0S2, HBEGF*, and *PMEPA1* (prostate transmembrane protein, androgen induced 1), which showed EC_50_ values for 25(OH)D below 150 nM. Thus, in healthy persons with a very high vitamin D status, a few genes may be directly affected by elevated 25(OH)D serum levels.

In addition to the control of the vitamin D status of healthy individuals through careful sun exposure and vitamin D_3_ or D_2_ supplementation, there are clinical settings, where supplementation with higher doses of 25(OH)D_3_ or 25(OH)D_2_ are recommended (52). These patients may reach, at least for a limited time, far higher 25(OH)D serum levels than healthy individuals. Moreover, 25(OH)D_3_ is used as a food supplement in animal farming, *e.g*., for accelerating the growth of chicken (53). Also in these settings elevated 25(OH)D_3_ serum levels may be reached. Thus, there are a few scenarios, in which larger parts of the vitamin D-dependent transcriptome may be affected by 25(OH)D supplementation.

Studying the transcriptome‘s sensitivity to treatments with vitamin D metabolites led to the interesting observation that vitamin D target genes can be distinguished in high, mid and low responders. This suggests that not all vitamin D target genes respond equally to a stimulation with a given concentration of a VDR ligand. High responding genes, the best known of which are *HBEGF* (54) and *G0S2* (55), get activated at already at 5-times lower levels than the average of all genes, while low responding genes, such as *LMNA* (56) and *STAB1* (48), need for their response up to 5-times higher 1,25(OH)_2_D_3_ concentrations than the mean. Interestingly, high responding genes tend to be primary targets that are directly regulated by activated VDR binding to enhancers in the vicinity of the gene's transcription start sites (57), while low responding genes are preferentially secondary targets that are regulated by transcription factors encoded by primary target genes (31). This adds a new characteristic to the description of vitamin D target genes, the mechanistic basis and physiological meaning of which needs to be further explored in the future.

For the main aim of this study, the comparison of the gene regulatory potential 25(OH)D_3_ and 25(OH)D_2_, it does not matter, if the observed effects on the PBMC transcriptome are explained either (i) by the enzymatic conversion of 25(OH)D into 1,25(OH)_2_D during the 4–24 h duration of stimulation phase, which then activates the VDR, (ii) by a direct binding of 25(OH)D to the VDR or (iii) a combination of both. The very low expression of the *CYP27B1* gene, in particular in relation to *CYP24A1* expression, in PBMCs of one person used in this study is representative for other individuals. This calls into question, whether there was enough 1α-hydroxylase activity to convert within 4 h a sufficient amount of 25(OH)D into 1,25(OH)_2_D, which then stimulated primary vitamin D target genes. For example, in order to reach a 1,25(OH)_2_D level of 10 nM, 1% of the 1,000 nM 25(OH)D pool need to be handled within 4 h. However, as it is typical for *in vitro* cell culture stimulation experiments, supra-physiological concentrations of 1,25(OH)_2_D_3_ and 25(OH)D are compared. In fact, the tight regulation of the 1,25(OH)_2_D_3_ level *in vivo via* the molecule's rapid degradation by the enzyme CYP24A1 (58), indicates that concentrations used *in vitro* most likely never occur *in vivo*.

In conclusion, 25(OH)D_3_ and 25(OH)D_2_ are equally potent in modulating the transcriptome of PBMCs and regulate the same set of vitamin D target genes as the most potent VDR ligand, 1,25(OH)_2_D_3_. However, in order to observe consequences of the gene regulatory potential of 25(OH)D, concentrations of 300 nM or higher need to be available. This is three times the recommended serum level, i.e., it does not apply to healthy individuals with a regular vitamin D status.

## Data Availability Statement

Fastq files of the raw data can be found at GEO with accession number GSE199273.

## Ethics Statement

The studies involving human participants were reviewed and approved by the Ethics Committee of the Northern Savo Hospital District had approved the study protocol (#9/2014). The patients/participants provided their written informed consent to participate in this study.

## Author Contributions

AH performed RNA-seq data analysis. CC did RNA isolation and RNA-seq library preparation. CV synthesized the tested compounds. AH and CC wrote the manuscript, which was reviewed by CV. All authors contributed to the article and approved the submitted version.

## Funding

Parts of this study were sponsored by Carbogen Amics, Ltd. CC was supported by the WELCOME2—ERA Chair European Union's Horizon2020 research and innovation program under Grant Agreement No. 952601.

## Conflict of Interest

This study received funding from Carbogen Amics, Ltd. The funder had the following involvement with the study: Providing vitamin D metabolites. The funder was not involved in the study design, collection, analysis, interpretation of data, the writing of this article or the decision to submit it for publication. The authors declare that the research was conducted in the absence of any commercial or financial relationships that could be construed as a potential conflict of interest.

## Publisher's Note

All claims expressed in this article are solely those of the authors and do not necessarily represent those of their affiliated organizations, or those of the publisher, the editors and the reviewers. Any product that may be evaluated in this article, or claim that may be made by its manufacturer, is not guaranteed or endorsed by the publisher.
