# Supplementary material for: Gene-Regulatory Potential of 25-Hydroxyvitamin D3 and D2
Source: Front Nutr. 2022 Jul 13;9:910601. doi: 10.3389/fnut.2022.910601 (PMC9330572; doi:10.3389/fnut.2022.910601)
Supplement: Supplementary file 3 [file Data_Sheet_1.PDF]

SUPPLEMENTARY DATA

SUPPLEMENTARY TABLE

Table S1: RNA-seq read alignment. Numbers of uniquely aligned reads of each of the 66 samples are indicated.

Table S2: Vitamin D target genes in PBMCs. In total, 553 differentially expressed genes were identified using the statistical test *glmTreat* with the thresholds FC > 1.5 at time points 4 and 8 h and FC > 2 at 24 h. Classification of the genes as primary, secondary, direct and indirect was retrieved from a reference dataset [31]. Raw RNA-seq counts and normalized gene expression with statistical metrics are found at GEO with accession number GSE199273.

SUPPLEMENTARY FIGURES

Fig. S1: Quality assessment of RNA-seq data *via* MDS. The dimensionality reduction technique MDS was used to inspect (on the basis of the top 500 protein coding genes) the underlying data structure and similarity between the 66 RNA-seq samples of this study. The two main drivers of gene expression changes in PBMCs are the cells' time-dependent distancing from their native transcriptome state (dimension 1) and the vitamin D metabolite concentration (dimension 2). Distances on the plot approximate the typical $\log_2$FC between the samples, *i.e.*, one unit represents a FC of 2.

Fig. S2: Genome-wide effects of treatment. MA plots show the global effects of the treatment of PBMCs with indicated concentrations of 1,25(OH)$_2D_3$, 25(OH)D_3 or 25(OH)D_2 for 4, 8 or 24 h. The expression change (*i.e.*, treatment versus control ($\log_2$FC)) for each gene is shown in relation to its mean expression level between the compared groups. The gene-wise statistical test for differential expression was carried out as a pairwise comparison of treated versus (time-matched) control (0.1% EtOH) samples by using *glmTreat* method, *i.e.*, testing for differential expression relative to an absolute FC > 1.5 at the early time points 4 and 8 h and absolute FC > 2 at 24 h. Significantly (FDR < 0.05) up- and down-regulated genes are highlighted in red and blue, respectively. The horizontal red lines indicate the applied testing threshold. MA plots of 6 further treatments are displayed in **Figs. 1A and 3A**.

Fig. S3: Relations between target genes at different treatment conditions. Upset plots visualize the relations of target genes that had been identified after 24 h treatment with different vitamin D metabolite concentrations before (**A**) and after filtering with a reference dataset [31] (**B**). Bar charts on the right indicate the total number of identified target genes per treatment.

Fig. S4: Categories of vitamin D target genes. Based on a reference dataset [31], the 206 common vitamin D target genes are categorized into groups of primary/secondary,

direct/indirect and up-regulated/down-regulated genes with indicated numbers of members. Sigmoidal fitting of the change in average absolute expression in function of concentration of 1,25$(OH)_2D_3$, 25$(OH)_3$ or 25(OH)D_2 allowed the determination of EC_{50} values. Standard errors of the EC_{50} estimates are indicated. No significant difference between the target gene categories was observed.

Fig. S5: Relations between target genes at different treatment times. Upset plots visualize the relations of target genes that had been identified after 4, 8 and 24 h treatment with 10 nM 1,25$(OH)_2D_3$, 1000 nM 25(OH)D_3 or 1000 nM 25(OH)D_2 before (**A**) and after filtering with a reference dataset [31] (**B**). Bar charts on the right indicate the total number of identified target genes per treatment.

Fig. S6: Relative expression of genes of vitamin D metabolizing enzymes. The mean of 1,25$(OH)_2D_3$-treated and untreated mRNA expression of the genes *DHCR7*, *CYP2R1*, *CYP27A1*, *CYP27B1* and *CYP24A1* of 12 individuals participating in the vitamin D intervention trial VitDHiD [50] is displayed in $\log_2$-scale. The individuals are ranked by increasing *CYP27B1* expression.
