# Supplementary figures and images for: Gene-Regulatory Potential of 25-Hydroxyvitamin D3 and D2
Source: Front Nutr. 2022 Jul 13;9:910601. doi: 10.3389/fnut.2022.910601 (PMC9330572; doi:10.3389/fnut.2022.910601)

### Fig. S1

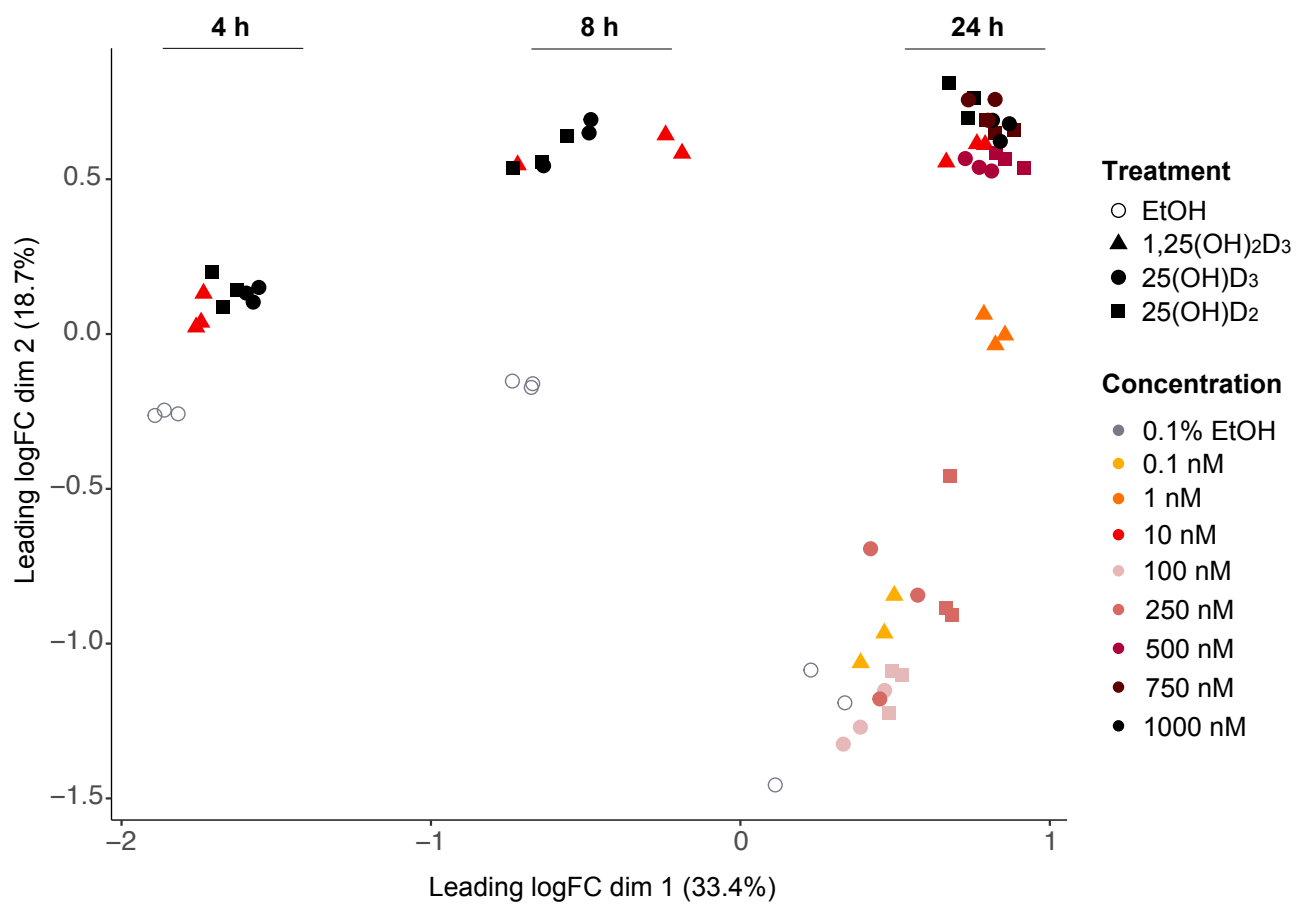

Supplement: Supplementary file 4 [file Image_1.pdf]

**Fig. S2**

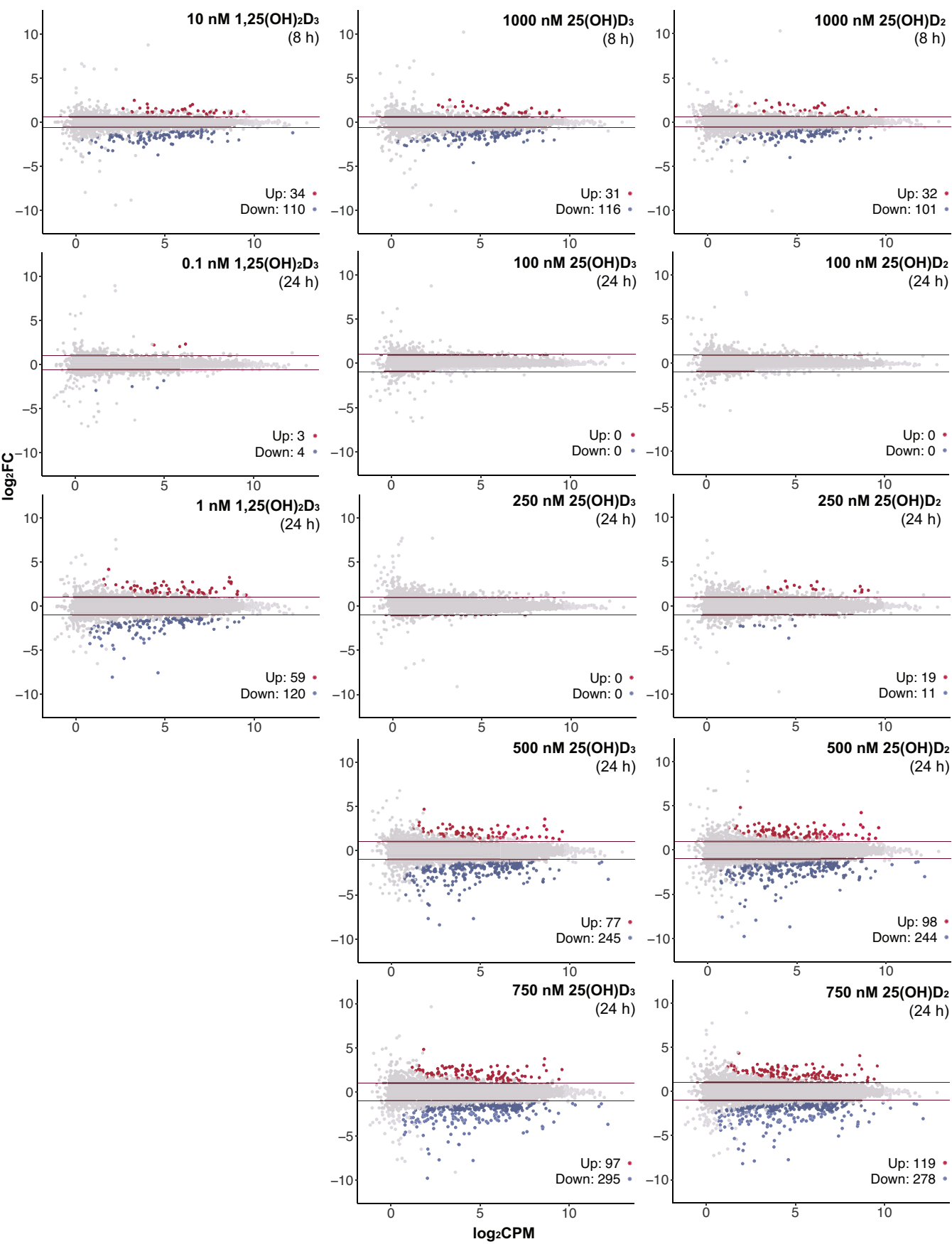

Supplement: Supplementary file 5 [file Image_2.pdf]

Fig. S3

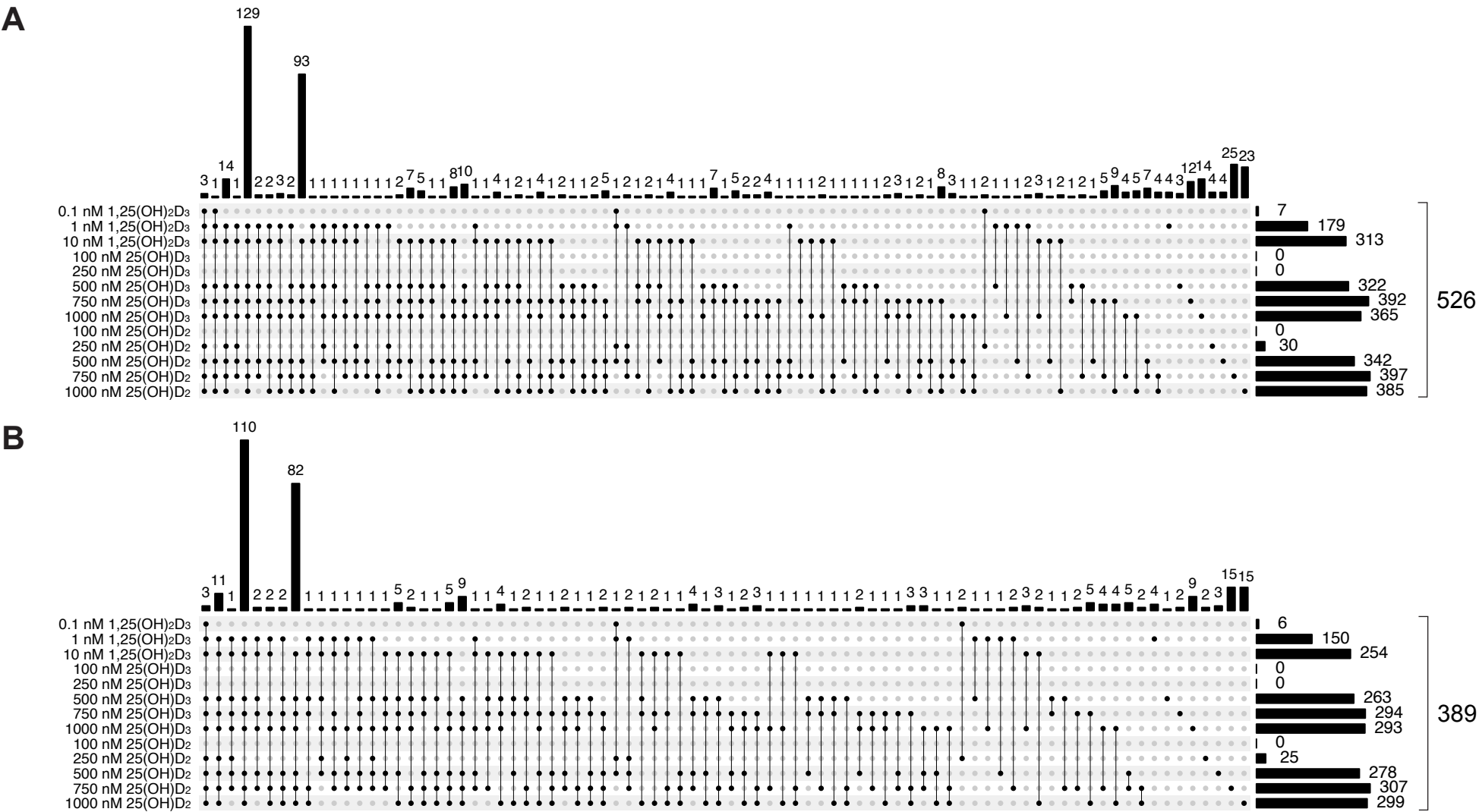

Supplement: Supplementary file 6 [file Image_3.pdf]

Fig. S4

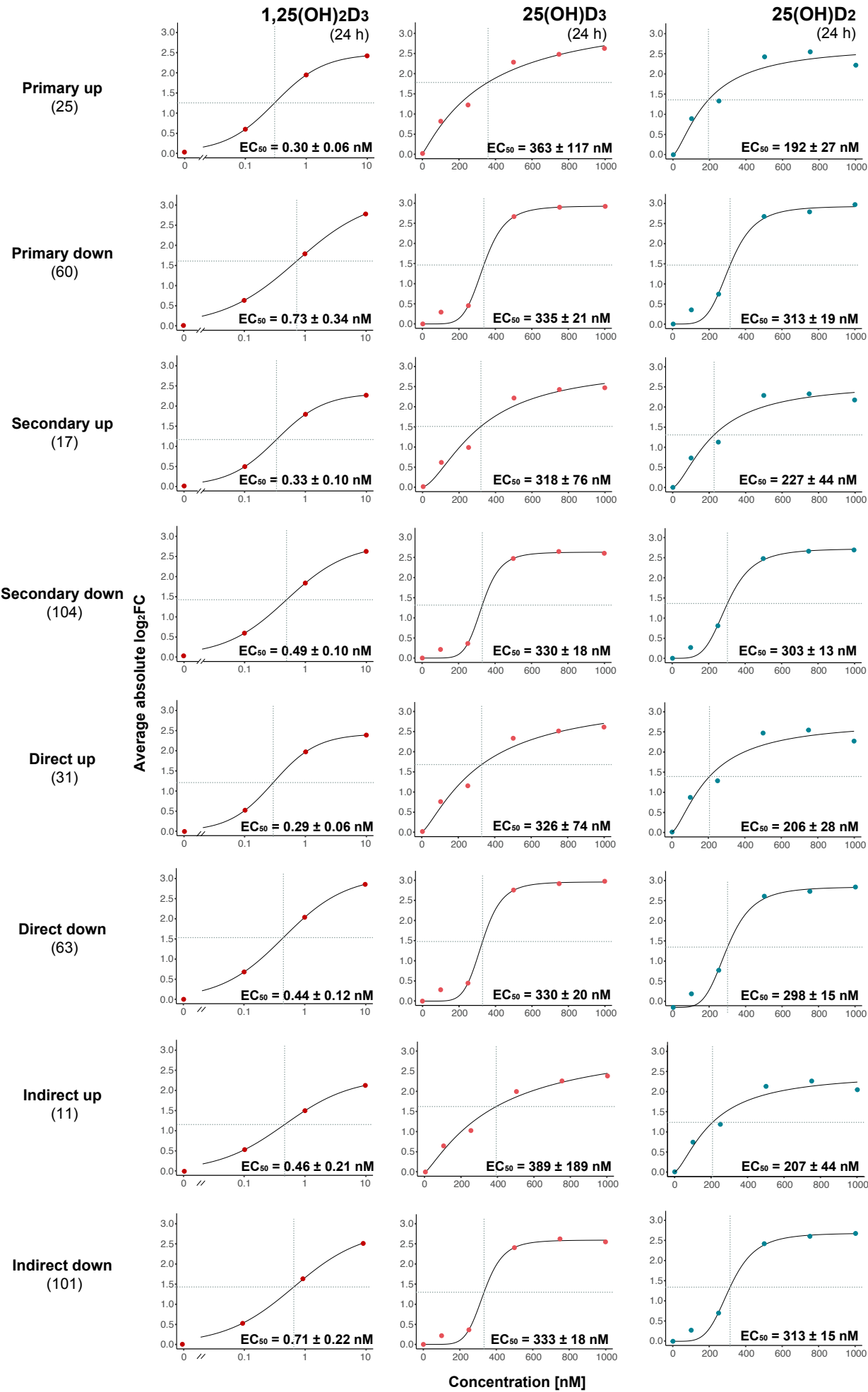

Supplement: Supplementary file 7 [file Image_4.pdf]

**A**

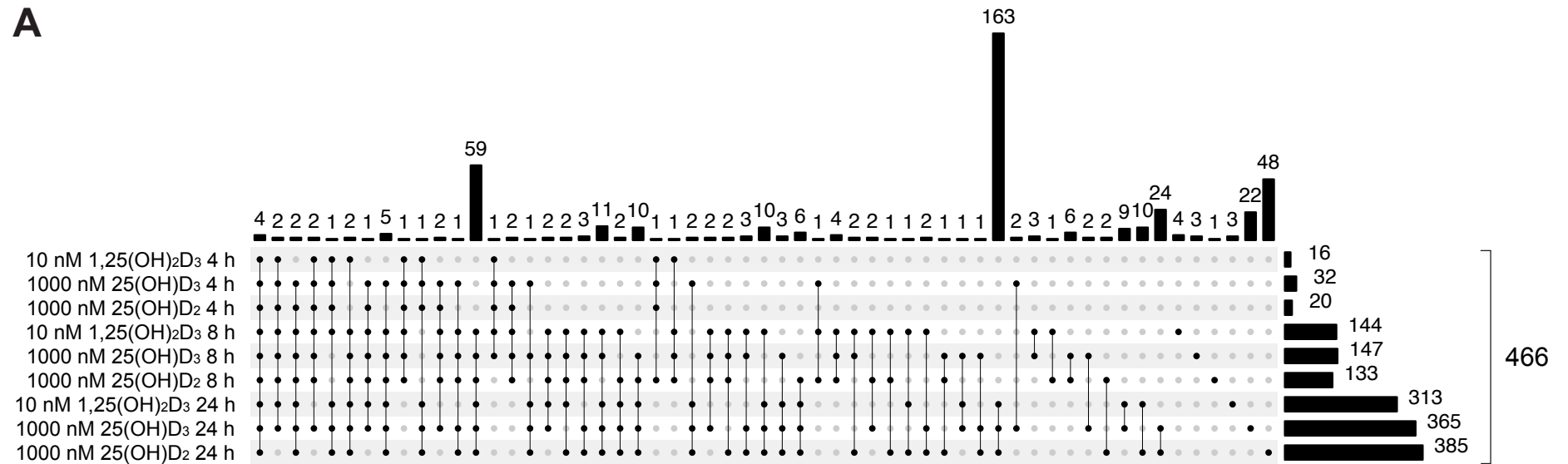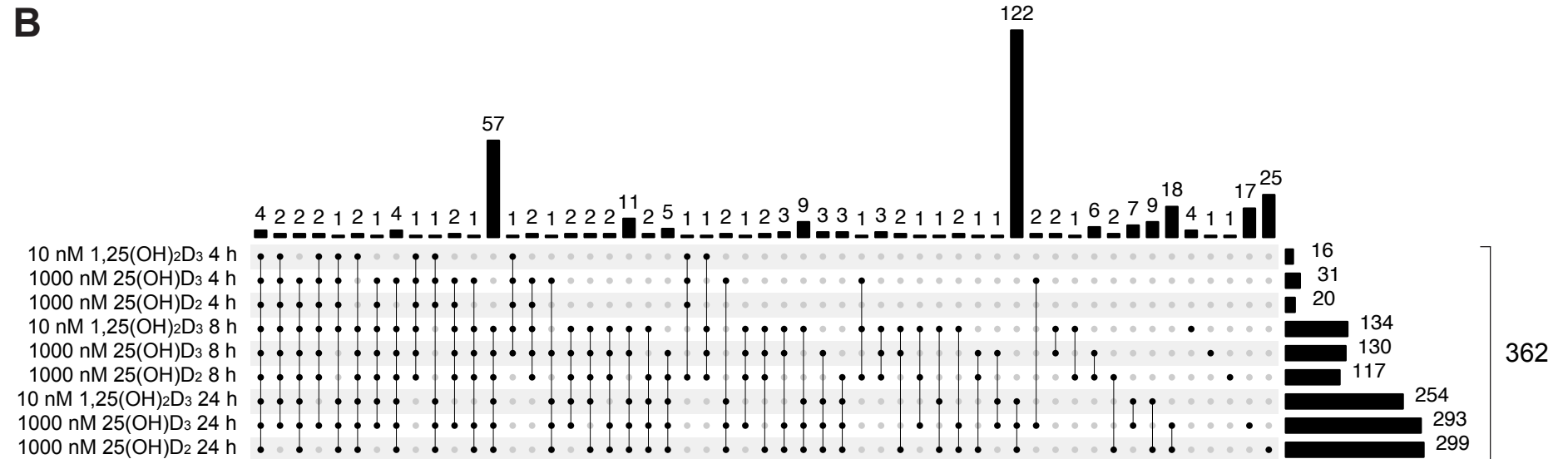

Supplement: Supplementary file 8 [file Image_5.pdf]

Fig. S6

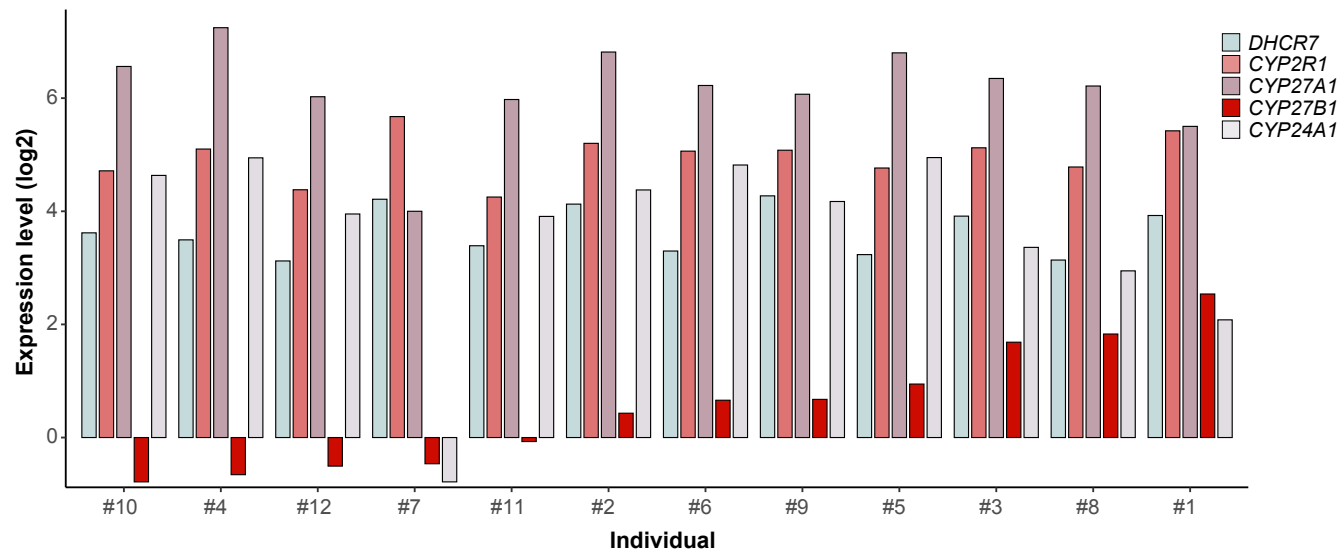

Supplement: Supplementary file 9 [file Image_6.pdf]
